# Large diurnal temperature range increases bird sensitivity to climate change

**DOI:** 10.1038/srep16600

**Published:** 2015-11-13

**Authors:** Michael Briga, Simon Verhulst

**Affiliations:** 1 Groningen Institute for Evolutionary Life Sciences, University of Groningen, 9747 AG Groningen, The Netherlands

## Abstract

Climate variability is changing on multiple temporal scales, and little is known of the consequences of increases in short-term variability, particularly in endotherms. Using mortality data with high temporal resolution of zebra finches living in large outdoor aviaries (5 years, 359.220 bird-days), we show that mortality rate increases almost two-fold per 1°C increase in diurnal temperature range (DTR). Interestingly, the DTR effect differed between two groups with low versus high experimentally manipulated foraging costs, reflecting a typical laboratory ‘easy’ foraging environment and a ‘hard’ semi-natural environment respectively. DTR increased mortality on days with *low* minimum temperature in the easy foraging environment, but on days with *high* minimum temperature in the semi-natural environment. Thus, in a natural environment DTR effects will become increasingly important in a warming world, something not detectable in an ‘easy’ laboratory environment. These effects were particularly apparent at young ages. Critical time window analyses showed that the effect of DTR on mortality is delayed up to three months, while effects of minimum temperature occurred within a week. These results show that daily temperature variability can substantially impact the population viability of endothermic species.

Climate change affects the abundance and distribution of populations through changes in both mean and variability of climatic variables^1^,^2^,^3^,^4^,^5^,^6^,^7^. When investigating climate variability, usually time scales of months or years are considered^8^,^9^,^10^,^11^, but climatic variability over much shorter timescales, typically days, has also changed in recent decades, at least on a regional scale^12^,^13^,^14^,^15^. For example, average diurnal temperature range (DTR), the difference between maximum and minimum temperature within one calendar day, has increased more than 2 °C since approximately the 1960’s in Mexico, Bolivia, Patagonia, Madagascar, Indonesia, central Russia and the Western Himalaya, while other areas have experienced up to equally large decreases, for example in north-eastern Canada, north and central Africa and the Eastern Himalaya^12^,^15^,^16^,^17^,^18^. Climate change is thus also associated with changes in temperature variability on short time scales.

DTR responses independent of mean temperature can occur following Jensen’s inequality^19^,^20^: when there are nonlinear associations between a system and its environment, mean system state will change in response to increased environmental variation even when the environment mean remains constant (Fig. 1). Increasing DTR has been shown to reduce population viability of ectotherms^21^,^22^,^23^,^24^,^25^, although the strength and direction of the effect can depend on the (mean) temperature^26^,^27^. Endotherms might also be susceptible to DTR but knowledge of DTR effects in endotherms is restricted to humans, where the elderly experience up to 3% increase in hospital admissions and 1% increase in mortality per 1 °C increase in DTR^28^,^29^,^30^,^31^,^32^,^33^. However, elderly humans behave very differently from endotherms in natural environments that are permanently exposed to natural variation in temperature. Thus the demographic and ecological consequences of changes in DTR in endotherms in natural environments remain unknown.

We used high-resolution (daily) mortality data to investigate the association between DTR and the survival of zebra finches (n = 476) housed in outdoor aviaries, and hence exposed to natural variation in temperature (Fig. 2 A,B). Our population has resided in captivity for generations, but the species is originally widely distributed in Australia. The natural variation in DTR in our aviaries is entirely within the natural range^34^. The minimum temperature (MinT) can be lower in the Netherlands than in Australia^35^, but in various areas of Australia zebra finches regularly experience MinT below zero^35^,^36^,^37^ and our results were still supported when conditions outside the zebra finches natural range were excluded.

The timeframe over which one investigates effects of climatic variables on mortality (or any other trait) can be chosen in different ways. For example one can arbitrarily choose to average climatic variables over one or two weeks before each day. However, such arbitrary choices may not reflect the timeframe over which the biological effects occur, and this approach implicitly makes the unlikely assumption that all days within the selected time interval have equal effects on the phenomenon that is studied. To resolve this issue we calculated the (weighted) time window over which climatic variables best correlated with mortality using a technique recently introduced by van de Pol and Cockburn^38^.

Data were collected over 5 years in the context of an experiment in which we manipulated environmental conditions during development (brood size) and in adulthood (foraging costs) in a 2 × 2 design with the primary aim to study effects on ageing and lifespan. Based on published results in ectotherms and humans^21^,^22^,^23^,^24^,^28^,^29^,^30^,^31^,^32^,^33^, we hypothesized, before analyzing the data, that large DTR could increase mortality. We further hypothesised that the strength of a DTR effect may depend on current or past environmental quality. Large broods are a poor developmental environment that causes pervasive negative effects during adulthood in many species^39^,^40^,^41^,^42^,^43^, and hence this manipulation allows us to investigate whether effects of DTR depend on phenotypic quality. In laboratory environments no effort has to be made to obtain food. In contrast, in more natural environments animals experience foraging costs. The foraging cost experiment thus allows us to compare DTR effects on mortality between a typical ‘easy’ laboratory foraging environment, with low foraging costs, and a ‘hard’ semi-natural foraging environment, with high foraging costs. Our expectation is that, since DTR represents a challenge, DTR will have more pronounced effects on mortality in animals that experience(d) poor quality environments.

## Results

The climatic data are shown in Fig. 2. Estimating the time frame over which the climatic variables best explained mortality showed that the effects were most pronounced the day preceding the event (i.e. the survival or death of an individual), accounting for 77% and 15% of the weight for MinT and DTR respectively (Fig. 3). The time window over which MinT affected mortality was much shorter than DTR (8.9 < ΔAICc < 12.5, see methods for details on test). For MinT, (almost) 80% of the effect was captured the day before the event, while reaching 80% for DTR required 3 months (Fig. 3). Thus MinT had an immediate effect on bird mortality in comparison with DTR for which the effect was delayed.

DTR and MinT both affected mortality but in an interaction, which received strong support: in both foraging environments all models within 4 AICc of the best fitting model contained the interaction between DTR and MinT (Table 1). However, the sign of the interaction term depended on foraging environment (Supplementary Table S3), which we discuss in more detail below. This three-way interaction (Treat*DTR*MinT) is well supported since it was included in all 14 best fitting models (Supplementary Table S3). In the case of the best fitting model this interaction had a *X* ^*2*^= 10.411, p = 0.0013, and removing it from the best fitting model decreased model fit by 9.2 AICc. Furthermore, all selected models contained an interaction between age and DTR, indicating a changing DTR effect with increasing age (Table 1; Supplementary Table S2). Because in natural populations most birds are young, we here focus the presentation on young birds (but note that models in Table 1 are based on the complete data set). Details of age-specific changes are discussed in section 4 of the supplementary material.

In the easy foraging environment, the effect of DTR was most pronounced on cold days (Fig. 4A, Supplementary Fig. S4A): birds experienced an up to ten-fold increase in mortality over the DTR range in our dataset. Evidence for this is robust since all selected models (Table 1) included DTR and the interaction between DTR and MinT. Thus, in the easy foraging environment, we found that large DTR increased mortality on cold days, but not on warm days.

In the semi-natural foraging environment the evidence for an interaction between DTR and MinT was also robust: excluding the interaction decreased model fit with at least 3.3 AICc and models without the interaction all had weights ≤0.04 (Table 1). Note however that coefficients for the DTR*MinT interaction were in the opposite direction compared to the easy environment (Table 1). Indeed large DTR increased mortality on warm days, but not on cold days (Fig. 4B, Supplementary Fig. S4B). Note that for DTR the coefficient in the best fitting model is larger in the semi-natural than in the easy environment on days with MinT of 6 °C, which is the mean MinT at our study location (2.04 vs. 1.36 respectively in the best fitting model). This implies an increase of 1 °C DTR has a stronger effect in the semi-natural than in the easy foraging environment (increases in mortality rate per °C DTR of 104% vs. 36% respectively). Thus in the semi-natural foraging environment large DTR increased mortality on warm days but not on cold days, opposite to the pattern in the easy foraging environment.

While there is strong evidence that growing up in large broods negatively affects lifespan in the semi-natural environment (Table 1), there is little support for the hypothesis that brood size manipulation affects vulnerability to DTR in either environment (Table 1).

DTR varied seasonally (Fig. 2), and the association between DTR and mortality can therefore be confounded with other climatic variables with similar seasonal variation as DTR. We captured the seasonal variation of climatic variables by adding photoperiod as a covariate to the best models of Table 1 and Supplementary Tables S2, S3 and S4. Photoperiod was in no case significant (0.23 < *X*^2^ < 2.29, 0.13 <p < 0.63), never improved the model fit (0.3 < ΔAICc < 6), and the effect on the model coefficients of DTR, MinT or their interaction was negligible. We therefore conclude that DTR contributes to mortality independently of the seasonal variation of other (climatic) variables.

## Discussion

A large DTR substantially increased mortality rate and this effect was modulated by minimum temperature, age and environment, but not by developmental conditions. That DTR affects mortality is relevant because climate change is associated with changes in temperature variability on short time scales, i.e. days^12^,^13^,^14^. Yet, to our knowledge this is the first study on the mortality consequences of changes in DTR in a non-human endotherm. Our study shows that changes in DTR can potentially pose a threat to the population viability of endotherms and that this threat is most apparent in semi-natural environments. Note however that our study exploited natural variation in DTR and hence we cannot exclude the possibility that other climatic variables contributed to the observed patterns.

Our results indicate that responses to changes in climate variability differ considerably between laboratory and semi-natural environments, in that foraging costs determined the temperature range at which birds are most susceptible to large DTR. DTR increased mortality more on days with *low* minimum temperature in an easy foraging environment, but more on days with *high* minimum temperature in a semi-natural foraging environment. A possible reason for such environment dependent effects is heat stress, which in this experiment can arise in the semi-natural foraging environment because of the combination of high temperatures with unavoidable heat production through increased foraging effort, which can have major effects on bird behaviour and physiology^44^. Muscular exercise decreases heat tolerance because it generates heat which needs to be dissipated to avoid for example mitochondrial and immune dysfunction, DNA damage, organ failure and even death^45^,^46^,^47^,^48^,^49^,^50^,^51^. Such interaction effects are important when estimating the biological consequences of climate change^52^. Our results indicate that climate change experiments in laboratory conditions may not simply underestimate impacts of climate change, but may provide completely contradictory results to natural conditions. Since climate change is associated with increases in minimum temperatures^13^,^14^, our result also suggests that the DTR effect in natural populations may become more important in a warming world.

Associations between DTR and survival changed with age (Fig. S3). The dependence of the DTR effect on age may be due to individual heterogeneity in combination with selective disappearance: individuals that are sensitive to large DTR die and thus only birds that are relatively DTR insensitive remain at old age. It is worth noting that in natural populations the majority of individuals are young^53^ and hence natural populations are likely to be more susceptible to DTR effects than our relatively protected study population.

We estimated the time window over which climate variables affected mortality, and found this to differ considerably between climatic variables, with MinT having a more immediate effect than DTR. The contrast between these time windows indicates that these climatic variables affect mortality through mechanisms that operate on different time scales. That MinT affected mortality on a short time scale is likely to reflect limits on the instantenous capacity to generate heat. We are less certain regarding the mechanism through which DTR affects mortality. However, birds adjust physiologically their energy allocation to ambient temperatures within days^54^,^55^,^56^,^57^ and short term temperature variation increases daily energy expenditure^58^. The delayed DTR effect may thus reflect increased vulnerability due to the cumulative physiological acclimatization costs when DTR is high for a prolonged period.

In conclusion, our results show that DTR strongly affects avian mortality. DTR effects on mortality have previously been demonstrated in one other endotherm, humans, but our finding of an almost two-fold increase in mortality per °C DTR substantially exceeds the 1–3% increase in hospital admissions and 1% increase in mortality found in humans^28^,^29^,^30^,^31^,^32^,^33^. We note however that time windows over which DTR affects human mortality have to our knowledge not been quantified, and by definition such an analysis would yield stronger DTR effects than hitherto reported. In humans, large DTR is associated with cardiovascular and respiratory dysfunctions, causing increased hospital admissions and mortality^28^,^29^,^32^,^33^ but whether the same mechanisms causes the DTR effects in birds remains to be established. Understanding the physiological mechanisms involved in the DTR effect is of interest in its own right, and may help predict which and when populations are most at risk. However, regardless of the underlying physiological mechanisms, our results, together with those found in humans, show that DTR effects are important for survival and hence for understanding and predicting population responses to climate change^59^.

## Methods

### Birds and housing

All birds used in this study were reared and housed at the University of Groningen, the Netherlands (53° 13′ 0” N/6° 33′, 0” E). Birds were bred indoors in single housed pairs housed in 80 × 40 x 40 cm (I × h × d) cages with two perches, a wooden nestbox and abundant nesting material (hay). Food (tropical seed mixture), water, grit and cuttlebone were provided ad libitum. In addition the birds received one teaspoon of fortified canary food (“eggfood”, by Bogena, Hedel, the Netherlands) 3 times a week, until hatching of the first chick. Birds were cross-fostered when the oldest chick in a brood was 5 days old to broods that were either small (2 young, sometimes 3) or large broods (6 young, sometimes 5 or 7). Birds reared in a large brood attained lower body mass during growth and this effect persisted into adulthood (Briga *et al.* submitted). Young were removed from the parental cage when 35 days old and housed in indoor aviaries until they were entered in the experiment at 3–4 months of age.

Adults were housed in eight large outdoor aviaries (L × H × W 310 × 210 × 150 cm) and subject to a foraging cost manipulation as described previously^60^. Briefly, in each aviary a food box was attached to the ceiling, with holes in the sides from which food (tropical seed mixture) could be obtained. In the easy foraging environment (4 aviaries) the food box has perches beneath the holes, while in the hard foraging environment these were removed (also 4 aviaries), forcing birds to fly and hover for seeds. Water (for drinking and bathing), grit and cuttlebone were provided ad libitum. In addition the birds received 1.25 g of fortified canary food (“eggfood”, by Bogena, Hedel, the Netherlands) per individual per week in three portions given on different days.

Each aviary contained 15–30 birds of one sex (4 aviaries of each sex). To maintain numbers within a limited range, new birds were periodically added to replace dead birds. The first batch was 3–24 months old when the experiment started and variation in age when entering the experiment (‘AgeStart’) was therefore included as variable in all analyses. The first batch was kept in similar housing as in the experiment until the experiment started.

The foraging experiment was conducted from Dec 9^th^ 2007 till Jan 1^st^ 2013. During this period, 478 birds were entered in the experiment of which 285 died a natural death and 7 died an accidental death. In all analyses, accidental deaths and birds still alive were censored, but treating accidental deaths as natural deaths did not change the conclusions (results not shown).

All methods and experimental protocols were carried out under the approval of the Animal Experimentation Ethical Committee of the University of Groningen, license 5150A. All methods were carried out in accordance with these approved guidelines.

### Temperature data

**Figure 1:**
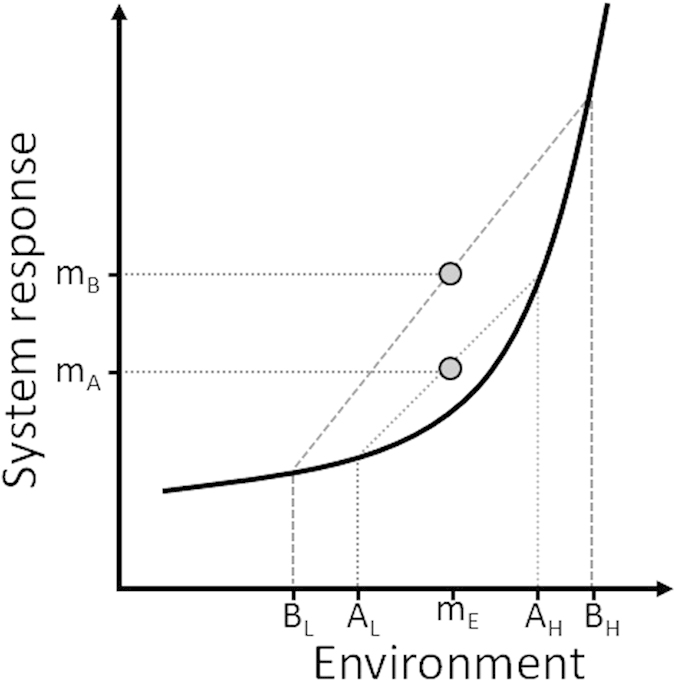
Illustration of Jensen’s inequality in a convex environment-system response scenario. Two environments (**A**,**B**) with the same mean state m_E_ can both be in one of two states (A_L_ & A_H_; B_L_ & B_H_) with equal frequency but with different ranges (range_A_<range_B_). It can be seen that because of the convex pattern the mean system response differs between environments A and B (m_A_<m_B_) despite A and B having the same average.

**Figure 2:**
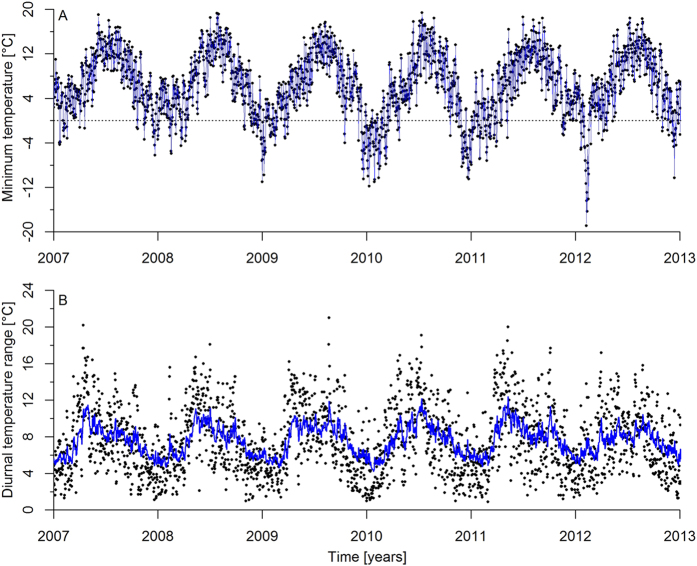
Minimum temperature (**A**) and diurnal temperature range data (**B**) during the study period. Black dots represent the actual data, while blue lines depict the weighted data that are used in the analyses (Table 1) based on the weight functions in Fig. 3. Dotted horizontal line is a reference line at MinT = 0 °C.

Temperature data (Fig. 1) were collected at the weather station of Eelde, approximately 7 km from the aviaries (http://www.knmi.nl/klimatologie/), where temperature was recorded 1.5 m above ground, every hour with accuracy of 0.1 °C. DTR is the difference between maximum (MaxT) and minimum (MinT) temperature within one day. Both MinT and DTR measured at the weather station correlate well with the measurements at the aviaries (N = 1196, r = 0.96 and 0.83 for MinT and DTR respectively; Supplementary Fig. S2).

Apparent effects of DTR on mortality could instead be caused by minimum or maximum temperature, because DTR will be higher when either minimum or maximum temperature has an extreme value. To resolve this issue, we included MinT as a covariate in addition to DTR in all analyses and also tested the interaction between MinT and DTR. Alternatively, we could have included MaxT instead of MinT. However, DTR = MaxT – MinT, and thus when MinT and DTR are given the corresponding MaxT is known. Hence having DTR in the model with either MinT or MaxT is mathematically equivalent. To confirm this point we reran the best fitting model in Table 1 with MaxT instead of MinT which as expected confirmed the importance of DTR on mortality.

### Statistical analyses

Survival was analysed using the counting process formulation of the Cox proportional hazard (CPH) model^61^,^62^,^63^ in R^64^, version 3.0.1 with the function ‘coxph’ of package survival^65^, version 2.37-4. The counting process formulation allows the coefficient to be estimated at each time point and thus time-dependent covariates, such as minimum temperature, DTR and age can be included. Time was portioned into daily intervals for all analyses.

**Figure 3:**
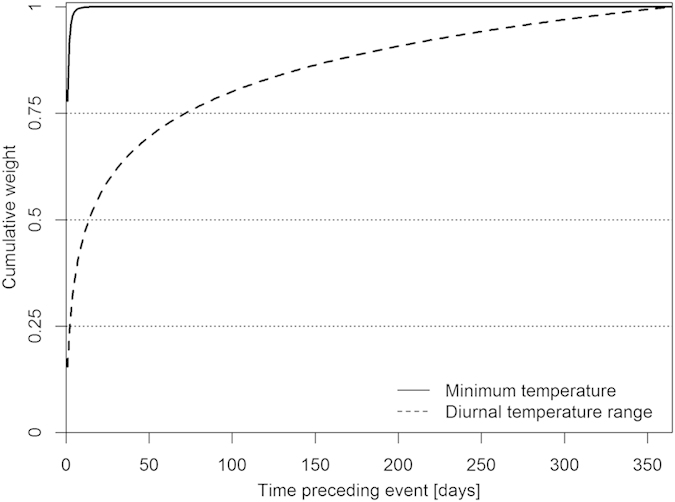
Cumulative weight functions (Weibull fits) that were best supported by the data. X-axis refers to time before the event (i.e. either survival or death of an individual) in days. Y-axis shows the cumulative weight or “influence” of the climatic variable on the event. For example for MinT, weights add up to 100% within 5 days, which means that all the minimum temperature of the 5 days prior to the event determine event outcome. For DTR the effect is delayed: 100% of the weight needs more than 3 months to accumulate. Horizontal dotted lines are reference lines at given weights.

**Figure 4:**
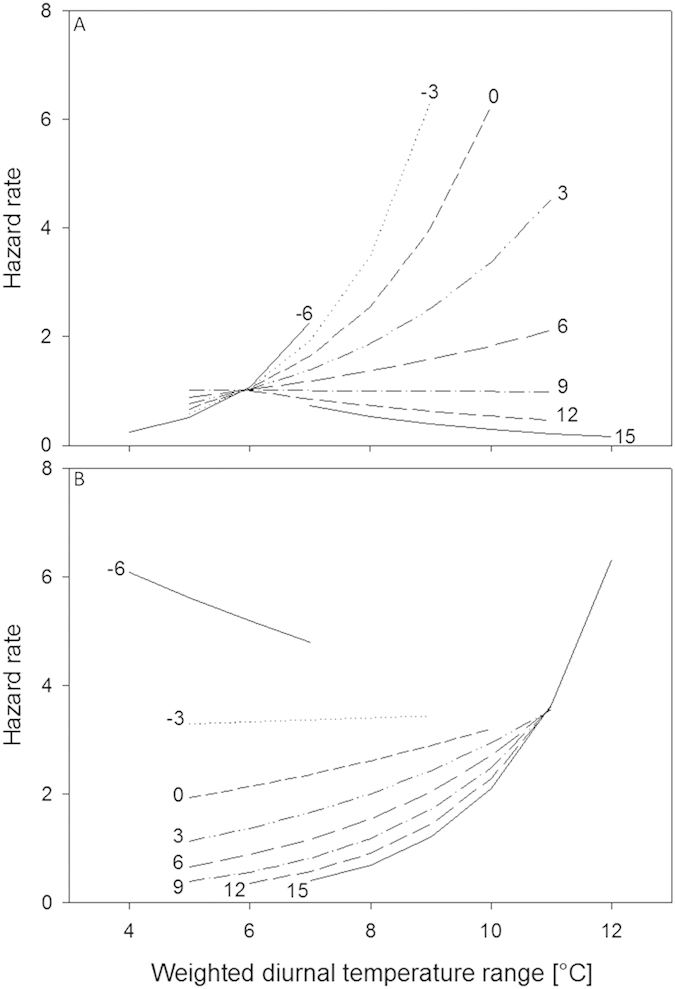
Hazard rate in relation to minimum temperature and diurnal temperature range (DTR) in easy (**A**) and semi-natural (**B**) foraging environment for the best fitting model of Table 1. Fitted lines represent hazard rates for different weighted minimum temperatures (temperatures plotted at line ends) calculated for individuals with a study age at start of 1 year old (population mean) and 0.36 years in study (at which 90% of the population is alive). Lines cover 95% of the data range. See Supplementary Fig. S4 for a contour plot with data distribution and hazard rates.

When analysing effects of climatic variables on system responses, the time window over which the climate variable affect system response needs to be identified. However, this time is usually not known. Should the temperature be quantified as a (weighted) mean over the preceding day, week or month? To resolve this, we identified the time window over which each climate variable affected survival using a flexible time window approach^38^. In brief, this method uses a maximum-likelihood optimization procedure to estimate a weight function over a time window that creates weighted temperature variables that best describe the variation in mortality data. As weight function we used a three-parameter Weibull function. Weight functions may differ between treatments and between climatic variables, and we thus estimated weight functions for each climatic variable separately. To estimate the strength of the difference in time windows between climatic variables, we used the weight function of one climatic variable to construct the other weighted climatic variable. We then compared the fit of this model relative to the fit of the model with the best fitting weight function. Model fits were compared using Akaike Information Criterion (AICc). Weight functions of the climatic variables did not differ between treatments (0.1 < ΔAICc < 0.5) and hence all analyses were carried out using the weight functions as in Fig. 3 in both treatments.

Except for the first batch to enter the adult phase of the experiment, other batches were housed indoors prior to being entered into the experiment. These birds were thus not exposed to the outdoor climatic variables before starting the foraging cost experiment and their mortality cannot be included in the survival analyses for the length of the period that the weighted climatic variable was calculated. Given the results of the time window analysis (Fig. 3), we excluded the first month of survival data after birds were entered in the foraging cost experiment. As a control we also ran the final model with (i) all data included and (ii) three months of data excluded, and both gave results that were consistent with those reported here (results not shown).

We used a model selection approach to find the model best supported by the data. To this end we followed Burnham and Anderson model selection approach^66^,^67^, based on Akaike Information Criterion (AICc) with the function ‘dredge’ of the package ‘MuMIn’^68^. In brief, this is a hypothesis-based approach that generates, given a global model, subset models that best fit the data. This makes it possible to assess model support for each hypothesis tested. Model support is shown here by ranking all subset models within 4 AICc of the best model fit. Weighted DTR and MinT were mean centered in all analyses.

The counting process formulation of the CPH model allows for non-proportionality by including the interaction between the main effect and time or age. Other assumptions of the CPH models were fulfilled as indicated by scaled deviance and martingale residual plots. Age was square-root transformed. Because, we found virtually no support for sex-specific mortality or for sex-specific DTR effects (see section 3 of the supplementary material), sexes were pooled in all analyses. Many random effects can potentially be included in these analyses: birth nest, genetic mother, genetic father, rear nest, rear mother, rear father, (birth) batch and aviary. We ran all models with aviary as random effect. We previously verified that including other random effects in CPH models did not improve the models (results not shown).

## Additional Information

**How to cite this article**: Briga, M. and Verhulst, S. Large diurnal temperature range increases bird sensitivity to climate change. *Sci. Rep.* **5**, 16600; doi: 10.1038/srep16600 (2015).

## Supplementary Material

Supplementary Information

## Figures and Tables

**Table 1:** Cox proportional hazard models in relation to climate, age and experimental treatments.

Easy foraging	Temperature variables [°C]			Age related correction variables [Years]				Developmental variables			Random term	Model Fit			
environment Model	DTR	MinT	DTR*MinT	AgeStart	AgeStart*Age	DTR*Age	MinT*Age	Brood Size	Brood Size *DTR	Brood Size *Age	Aviary	df	AICc	ΔAICc	weight
1	1.36	0.89	0.953	1.25	NA	0.74	1.06	NA	NA	NA	+	10	1269	0.00	0.09
2	1.22	0.95	0.951	1.24	NA	0.83	NA	NA	NA	NA	+	9	1269	0.03	0.09
3	0.98	0.95	0.951	1.22	NA	NA	NA	NA	NA	NA	+	8	1270	0.65	0.07
4	1.40	0.88	0.952	1.69	0.81	0.73	1.07	NA	NA	NA	+	11	1270	1.35	0.05
5	1.24	0.95	0.950	1.56	0.85	0.83	NA	NA	NA	NA	+	10	1271	1.62	0.04
6	1.36	0.89	0.953	1.26	NA	0.74	1.06	0.99	NA	NA	+	11	1271	1.86	0.04
7	1.22	0.95	0.951	1.24	NA	0.83	NA	0.99	NA	NA	+	10	1271	1.89	0.04
8	1.38	0.89	0.953	1.27	NA	0.74	1.06	1.13	NA	0.91	+	12	1271	2.08	0.03
9	0.97	0.91	0.952	1.23	NA	NA	1.03	NA	NA	NA	+	9	1271	2.08	0.03
10	1.23	0.95	0.951	1.25	NA	0.83	NA	1.13	NA	0.91	+	11	1271	2.08	0.03
11	1.20	0.95	0.951	1.25	NA	0.83	NA	0.98	0.97	NA	+	11	1271	2.31	0.03
12	0.98	0.95	0.951	1.47	0.88	NA	NA	NA	NA	NA	+	9	1271	2.39	0.03
13	1.24	0.95	0.951	1.26	NA	0.82	NA	1.12	0.97	0.90	+	12	1272	2.59	0.03
14	0.98	0.95	0.951	1.22	NA	NA	NA	0.99	NA	NA	+	9	1272	2.59	0.03
15	1.35	0.89	0.953	1.26	NA	0.74	1.06	0.98	0.97	NA	+	12	1272	2.66	0.02
16	1.39	0.89	0.953	1.27	NA	0.73	1.06	1.12	0.97	0.91	+	13	1272	2.81	0.02
17	0.98	0.95	0.951	1.23	NA	NA	NA	1.13	NA	0.91	+	10	1272	3.12	0.02
18	1.40	0.88	0.952	1.70	0.81	0.73	1.07	0.99	NA	NA	+	12	1272	3.22	0.02
19	1.24	0.95	0.950	1.56	0.85	0.83	NA	0.99	NA	NA	+	11	1272	3.49	0.02
20	0.97	0.95	0.951	1.22	NA	NA	NA	0.98	0.97	NA	+	10	1272	3.53	0.02
21	1.41	0.88	0.952	1.63	0.84	0.73	1.06	1.12	NA	0.91	+	13	1273	3.66	0.01
22	0.97	0.91	0.951	1.52	0.86	NA	1.04	NA	NA	NA	+	10	1273	3.75	0.01
23	1.25	0.95	0.950	1.50	0.88	0.82	NA	1.12	NA	0.91	+	12	1273	3.85	0.01
24	1.22	0.95	0.951	1.57	0.85	0.83	NA	0.98	0.97	NA	+	12	1273	3.94	0.01
**Semi-natural environment**															
**Model**	**DTR**	**MinT**	DTR*MinT	**AgeStart**	AgeStart*Age	DTR*Age	MinT*Age	**Brood Size**	Brood Size *DTR	Brood Size *Age	**Aviary**	**df**	**AICc**	**ΔAICc**	**weight**
1	2.04	0.88	1.03	1.34	NA	0.50	1.05	1.15	NA	NA	+	11	1247	0.00	0.19
2	1.82	0.93	1.04	1.33	NA	0.56	NA	1.31	NA	0.91	+	11	1247	0.13	0.18
3	2.06	0.88	1.03	1.35	NA	0.49	1.05	1.31	NA	0.91	+	12	1247	0.21	0.17
4	1.76	0.93	1.04	1.04	1.22	0.58	NA	1.32	NA	0.90	+	12	1248	1.33	0.10
5	1.99	0.88	1.03	1.16	1.12	0.50	1.05	1.15	NA	NA	+	12	1249	1.70	0.08
6	1.99	0.88	1.04	1.09	1.18	0.51	1.05	1.32	NA	0.90	+	13	1249	1.70	0.08
7	1.79	0.93	NA	1.34	NA	0.55	NA	1.15	1.00	NA	+	10	1250	3.32	0.04
8	1.82	0.92	NA	1.35	NA	0.55	NA	1.30	0.99	0.91	+	11	1251	3.57	0.03

Data shown are coefficients (or NA for variables not in the model, and + for included random terms). All models within 4 AICc of the best model are shown, ordered by AICc. Model weights are relative to all fitted models. Note that model coefficients are hazard ratios: a hazard of 1 implies no effect and, for example, a hazard ratio of 1.25 for ‘AgeStart’ means that the hazard rate increases 25% per year. There is no main effect ‘age’ because it is included ifn the baseline mortality. AgeStart: Age at start of the foraging experiment in years.

## References

[b1] Coulson T. *et al.* Age, sex, density, winter weather, and population crashes in Soay sheep. Science 292, 1528–31 (2001). 11375487 10.1126/science.292.5521.1528

[b2] Jenouvrier S., Barbraud C. & Weimerskirch H. Effects of climate variability on the temporal population dynamics of southern fulmars. J. Anim. Ecol. 72, 576–587 (2003). 10.1046/j.1365-2656.2003.00727.x 30893965

[b3] Boyce M. S., Haridas C. V. & Lee C. T. & the NCEAS Stochastic Demography Working Group. Demography in an increasingly variable world. Trends Ecol. Evol. 21, 141–8 (2006). 16701490 10.1016/j.tree.2005.11.018

[b4] Parmesan C. Ecological and evolutionary responses to recent climate change. Annu. Rev. Ecol. Evol. Syst. 37, 637–669 (2006).

[b5] Van de Pol M. *et al.* Effects of climate change and variability on population dynamics in a long-lived shorebird. Ecology 91, 1192–204 (2010). 20462133 10.1890/09-0410.1

[b6] García-Carreras B. & Reuman D. C. Are changes in the mean or variability of climate signals more important for long-term stochastic growth rate? PLoS One 8, e63974 (2013). 23691131 10.1371/journal.pone.0063974 PMC3653831

[b7] Thompson R. M., Beardall J., Beringer J., Grace M. & Sardina P. Means and extremes: building variability into community-level climate change experiments. Ecol. Lett. 16, 799–806 (2013). 23438320 10.1111/ele.12095

[b8] Easterling D. R. *et al.* Climate extremes: observations, modeling, and impacts. Science 289, 2068–2074 (2000). 11000103 10.1126/science.289.5487.2068

[b9] Schar C. *et al.* The role of increasing temperature variability in European summer heatwaves. Nature 427, 332–336 (2004). 14716318 10.1038/nature02300

[b10] Rahmstorf S. & Coumou D. Increase of extreme events in a warming world. Proc. Natl. Acad. Sci. 108, 17905–17909 (2012). 22025683 10.1073/pnas.1101766108 PMC3207670

[b11] O’Gorman P. A. Contrasting responses of mean and extreme snowfall to climate change. Nature 512, 416–418 (2014). 25164753 10.1038/nature13625

[b12] Vose R. S., Easterling D. R. & Gleason B. Maximum and minimum temperature trends for the globe: An update through 2004. Geophys. Res. Lett. 32, L23822 (2005).

[b13] Solomon S. *et al.* Climate change 2007: The Physical Science Basis. Contribution of Working Group I to the Fourth Assessment Report of the Intergovernmental Panel on Climate Change. (Cambridge University Press, 2007).

[b14] Stocker T. F. *et al.* Climate Change 2013: The Physical Science Basis. Contribution of Working Group I to the Fifth Assessment Report of the Intergovernmental Panel on Climate Change. (Cambridge University Press, 2013).

[b15] Wang G. & Dillon M. E. Recent geographic convergence in diurnal and annual temperature cycling flattens global thermal profiles. Nat. Clim. Chang. 4, 988–992 (2014).

[b16] Yadav R. R., Park W. K., Singh J. & Dubey B. Do the western Himalayas defy global warming? Geophys. Res. Lett. 31, L17201 (2004).

[b17] Englehart P. J. & Douglas A. V. Changing behavior in the diurnal range of surface air temperatures over Mexico. Geophys. Res. Lett. 32, L01701 (2005).

[b18] Jhajharia D. & Singh V. P. Trends in temperature, diurnal temperature range and sunshine duration in Northeast India. Int. J. Climatol. 31, 1353–1367 (2011).

[b19] Jensen J. Sur les fonctions convexes et les inégalités entre les valeurs moyennes. Acta Math. 30, 175–193 (1906).

[b20] Ruel J. J. & Ayres M. P. Jensen’s inequality predicts effects of environmental variation. Trends Ecol. Evol. 14, 361–366 (1999). 10441312 10.1016/s0169-5347(99)01664-x

[b21] Raffel T. R. *et al.* Disease and thermal acclimation in a more variable and unpredictable climate. Nat. Clim. Chang. 3, 146–151 (2012).

[b22] Paaijmans K. P. *et al.* Temperature variation makes ectotherms more sensitive to climate change. Glob. Chang. Biol. 19, 2373–80 (2013). 23630036 10.1111/gcb.12240 PMC3908367

[b23] Clavijo-Baquet S. *et al.* Differential responses to thermal variation between fitness metrics. Sci. Rep. 4, 5349 (2014). 24954717 10.1038/srep05349 PMC5381537

[b24] Zeh J. A. *et al.* Constant diurnal temperature regime alters the impact of simulated climate warming on a tropical pseudoscorpion. Sci. Rep. 4, 3706 (2014). 24424082 10.1038/srep03706 PMC3892181

[b25] Levy O. *et al.* Resolving the life cycle alters expected impacts of climate change. Proc. R. Soc. B Biol. Sci. 282, 20150837 (2015). 10.1098/rspb.2015.0837 PMC4632613 26290072

[b26] Vasseur D. A. *et al.* Increased temperature variation poses a greater risk to species than climate warming. Proc. R. Soc. B Biol. Sci. 281, 20132612 (2014). 10.1098/rspb.2013.2612 PMC3924069 24478296

[b27] Bozinovic F. *et al.* The mean and variance of environmental temperature interact to determine physiological tolerance and fitness. Physiol. Biochem. Zool. 84, 543–52 (2011). 22030847 10.1086/662551

[b28] Liang W.-M., Liu W.-P. & Kuo H.-W. Diurnal temperature range and emergency room admissions for chronic obstructive pulmonary disease in Taiwan. Int. J. Biometeorol. 53, 17–23 (2009). 18989710 10.1007/s00484-008-0187-y

[b29] Lim Y.-H., Hong Y.-C. & Kim H. Effects of diurnal temperature range on cardiovascular and respiratory hospital admissions in Korea. Sci. Total Environ. 417-418, 55–60 (2012). 22281041 10.1016/j.scitotenv.2011.12.048

[b30] Kan H. *et al.* Diurnal temperature range and daily mortality in Shanghai, China. Environ. Res. 103, 424–31 (2007). 17234178 10.1016/j.envres.2006.11.009

[b31] Cao J. *et al.* Diurnal temperature range is a risk factor for coronary heart disease death. J. Epidemiol. 19, 328–332 (2009). 19749499 10.2188/jea.JE20080074 PMC3924102

[b32] Song G. *et al.* Diurnal temperature range as a novel risk factor for COPD death. Respirology 13, 1066–9 (2008). 18922144 10.1111/j.1440-1843.2008.01401.x

[b33] Tam W. W. S., Wong T. W., Chair S. Y. & Wong A. H. S. Diurnal temperature range and daily cardiovascular mortalities among the elderly in Hong Kong. Arch. Environ. Occup. Health 64, 202–6 (2009). 19864223 10.1080/19338240903241192

[b34] Plummer N., Lin Z. & Torok S. Trends in the diurnal temperature range over Australia since 1951. Atmos. Res. 37, 79–86 (1995).

[b35] Zann R. A. The Zebra Finch: a synthesis of field and laboratory studies. (Oxford University Press, 1996).

[b36] Kikkawa J. Seasonality of nesting by zebra finches at Armidale. Emu 80, 13–20 (1980).

[b37] Immelman K. Versuch einer ökologischen Verbreitungsanalyse beim australischen Zebrafinken, *Taeniopygia guttata castanotis* (Gould). J. Ornithol. 106, 415–430 (1965).

[b38] Van de Pol M. & Cockburn A. Identifying the critical climatic time window that affects trait expression. Am. Nat. 177, 698–707 (2011). 21508615 10.1086/659101

[b39] Lindström J. Early development in birds and mammals. Trends Ecol. Evol. 14, 343–348 (1999). 10441307 10.1016/s0169-5347(99)01639-0

[b40] Metcalfe N. B. & Monaghan P. Compensation for a bad start: grow now, pay later? Trends Ecol. Evol. 16, 254–260 (2001). 11301155 10.1016/s0169-5347(01)02124-3

[b41] Griffith S. C. & Buchanan K. L. Maternal effects in the zebra finch: a model mother reviewed. Emu 110, 251–267 (2010).

[b42] De Kogel C. Long-term effects of brood size manipulation on and sex-specific mortality of morphological development offspring. J. Anim. Ecol. 66, 167–178 (1997).

[b43] Boonekamp J. J., Mulder G. A., Salomons H. M., Dijkstra C. & Verhulst S. Nestling telomere shortening, but not telomere length, reflects developmental stress and predicts survival in wild birds. Proc. R. Soc. B Biol. Sci. 281, 20133287 (2014). 10.1098/rspb.2013.3287 PMC4024283 24789893

[b44] Du Plessis K. L., Martin R. O., Hockey P. A. R., Cunningham S. J. & Ridley A. R. The costs of keeping cool in a warming world: implications of high temperatures for foraging, thermoregulation and body condition of an arid-zone bird. Glob. Chang. Biol. 18, 3063–3070 (2012). 10.1111/j.1365-2486.2012.02778.x 28741828

[b45] Sawka M. N., Leon L. R., Montain S. J. & Sonna L. A. Integrated physiological mechanisms of exercise performance, adaptation, and maladaptation to heat stress. Compr. Physiol. 1, 1883–1928 (2011). 23733692 10.1002/cphy.c100082

[b46] Gamo Y. *et al.* Limits to sustained energy intake. XX. Body temperatures and physical activity of female mice during lactation. J. Exp. Biol. 216, 3751–61 (2013). 23788704 10.1242/jeb.090308

[b47] Jimenez C. *et al.* Immune function during and after 60 min of moderate exercise wearing protective clothing. Aviat. Sp. Environ. Med. 79, 570–576 (2008). 10.3357/asem.2226.2008 18581940

[b48] Paul C., Teng S. & Saunders P. T. K. A single, mild, transient scrotal heat stress causes hypoxia and oxidative stress in mouse testes, which induces germ cell death. Biol. Reprod. 80, 913–919 (2009). 19144962 10.1095/biolreprod.108.071779 PMC2709966

[b49] Walsh N. P. & Whitham M. Exercising in environmental extremes: A greater threat to immune function? Sport. Med. 36, 941–976 (2006). 10.2165/00007256-200636110-00003 17052132

[b50] Yan Y. E., Zhao Y. Q., Wang H. & Fan M. Pathophysiological factors underlying heatstroke. Med. Hypotheses 67, 609–617 (2006). 16631316 10.1016/j.mehy.2005.12.048

[b51] Speakman J. R. & Król E. Maximal heat dissipation capacity and hyperthermia risk: Neglected key factors in the ecology of endotherms. J. Anim. Ecol. 79, 726–746 (2010). 20443992 10.1111/j.1365-2656.2010.01689.x

[b52] Parmesan C. *et al.* Beyond climate change attribution in conservation and ecological research. Ecol. Lett. 16, Suppl 1, 58–71 (2013). 23679010 10.1111/ele.12098

[b53] Jones O. R. *et al.* Diversity of ageing across the tree of life. Nature 505, 169–73 (2014). 24317695 10.1038/nature12789 PMC4157354

[b54] Swanson D. L. & Olmstead K. L. Evidence for a proximate influence of winter temperature on metabolism in passerine birds. Physiol. Biochem. Zool. 72, 566–75 (1999). 10521324 10.1086/316696

[b55] Bouwhuis S., Sheldon B. C. & Verhulst S. Basal metabolic rate and the rate of senescence in the great tit. Funct. Ecol. 25, 829–838 (2011).

[b56] Vézina F., Jalvingh K. M., Dekinga A. & Piersma T. Acclimation to different thermal conditions in a northerly wintering shorebird is driven by body mass-related changes in organ size. J. Exp. Biol. 209, 3141–3154 (2006). 16888062 10.1242/jeb.02338

[b57] Swanson D. L. Are summit metabolism and thermogenic endurance correlated in winter-acclimatized passerine birds? J. Comp. Physiol. B 171, 475–481 (2001). 11585259 10.1007/s003600100197

[b58] Pendlebury C. J. Variation in temperature increases the cost of living in birds. J. Exp. Biol. 207, 2065–2070 (2004). 15143140 10.1242/jeb.00999

[b59] Jenouvrier S. Impacts of climate change on avian populations. Glob. Chang. Biol. 19, 2036–57 (2013). 23505016 10.1111/gcb.12195

[b60] Koetsier E. & Verhulst S. A simple technique to manipulate foraging costs in seed-eating birds. J. Exp. Biol. 214, 1225–9 (2011). 21430197 10.1242/jeb.050336

[b61] Cox D. Regression models and life-tables. J. R. Stat. Soc. Ser. B-Statistical Methodol. 34, 187–220 (1972).

[b62] Andersen P., Borgan O., Gill R. & Keiding N. Statistical models based on counting processes. (Springer-Verlag, 1993).

[b63] Therneau T. & Grambsch P. Modeling survival data: extending the Cox model. (Springer-Verlag, 2000).

[b64] Core Team. R. R: A language and environment for statistical computing. (2014) Available at: http://www.r-project.org. (Accessed: 28^th^ April 2015).

[b65] Therneau T. A. Package for Survival Analysis in S. R package version 2.37-4. (2013) Available at: http://cran.r-project.org/package=survival. (Accessed: 28^th^ April 2015).

[b66] Burnham K. & Anderson D. Model selection and multimodel inference: A practical information-theoretic approach. (Springer-Verlag, 2002).

[b67] Burnham K. P., Anderson D. R. & Huyvaert K. P. AIC model selection and multimodel inference in behavioral ecology: Some background, observations, and comparisons. Behav. Ecol. Sociobiol. 65, 23–35 (2011).

[b68] Barton K. Package ‘MuMIn’. Model selection and model averaging based on information criteria. (2013) Available at: http://cran.r-project.org/web/packages/MuMIn/index.html. (Accessed: 28^th^ April 2015).

