# Supplementary material for: Large diurnal temperature range increases bird sensitivity to climate change
Source: Sci Rep. 2015 Nov 13;5:16600. doi: 10.1038/srep16600 (PMC4643245; doi:10.1038/srep16600)

**Supplementary information to ‘Large diurnal temperature range increases bird sensitivity to climate change’**

Michael Briga & Simon Verhulst

Groningen Institute for Evolutionary Life Sciences, University of Groningen, 9747 AG Groningen, The Netherlands

**Supplementary material 1: Information on temperature variables**

Supplementary Table S1: Summary statistics of weather variables used in this analysis.

|  | **MinT** | | **DTR** | |
| --- | --- | --- | --- | --- |
| **Year** | Mean | SD | Mean | SD |
| 2007 | 7.3 | 5.0 | 7.4 | 3.6 |
| 2008 | 6.8 | 5.6 | 7.5 | 3.6 |
| 2009 | 6.3 | 5.8 | 7.7 | 3.7 |
| 2010 | 4.6 | 7.0 | 7.7 | 3.8 |
| 2011 | 6.5 | 5.6 | 7.9 | 4.0 |
| 2012 | 6.1 | 6.3 | 7.4 | 3.3 |
|  |  |  |  |  |
|  |  |  |  |  |

Supplementary Figure S1: Diurnal temperature range data plotted against minimum temperature. Grey line shows correlation (r=0.51).

Supplementary Figure S2: Consistency between temperatures measured at the nearby weather station at Eelde and at the aviaries. Correlations are strong with r=0.96 and 0.83 for MinT and DTR respectively. Diagonal line shows x=y, grey line shows the fitted regression line. Note that we used weather station data over aviary data because of missing climate data for measurements at the aviaries.

**Supplementary material 2: Model support for foraging environment specific DTR effects.**

This analysis consists of 3 steps:

1. Model support for age specific associations between DTR and bird mortality (table S2). We thus analysed these patterns for young (<median age) and old birds (>median age) separately.
2. In young birds there is strong support for treatment specific effects of DTR (table S3),
3. Old birds show treatment specific associations between DTR and bird mortality while DTR overall has a positive effect on survival (table S4).

Supplementary Table S2: Associations between DTR and bird mortality weaken with age (as indicated by the grey column). Table shows model support based on AICc criteria, including all model fits on data within 4AICc of the best model. Table shows model support based on AICc criteria, including all model fits on data within 4AICc of the best model. The best model fit for each treatment is given in the top row. For fitting variables coefficients are given. Non fitting variables have coefficient NA. Grey columns emphasize the variables to use for the take home message. Note that these are a Cox proportional hazards models and model coefficients are thus hazard ratios. A hazard ratio of one implies no effect and for example a hazard ratio of 1.35 for ‘AgeStart’ means that the hazard rate increases 35% per year. Note that there is no main effect age since it is included in the baseline mortality curve. AgeStart: Age at start of the foraging experiment in years.

| **All data Ages** | **Temperature variables [°C]** | | | **Foraging treatment variables [Hard]** | | | | **Age related correction variables [Years]** | | | | | **Random term** | **Model Fit** | | | |
| --- | --- | --- | --- | --- | --- | --- | --- | --- | --- | --- | --- | --- | --- | --- | --- | --- | --- |
| **Model** | **DTR** | **MinT** | **DTR*MinT** | **Treat** | **Treat*DTR** | **Treat*MinT** | **Treat*DTR*MinT** | **AgeStart** | **AgeStart*Age** | **DTR*Age** | **MinT*Age** | **Treat*Age** | **Aviary** | **df** | **AICc** | **ΔAICc** | **weight** |
| 1 | 1.81 | 0.88 | NA | 0.96 | 0.78 | NA | NA | 1.35 | NA | 0.58 | 1.07 | NA | + | 7 | 2716 | 0.00 | 0.12 |
| 2 | 1.77 | 0.88 | 0.99 | 0.73 | 0.85 | 0.99 | 1.04 | 1.34 | NA | 0.59 | 1.06 | NA | + | 10 | 2717 | 0.31 | 0.11 |
| 3 | 1.80 | 0.88 | NA | 0.92 | 0.81 | 0.98 | NA | 1.35 | NA | 0.58 | 1.06 | NA | + | 8 | 2718 | 1.49 | 0.06 |
| 4 | 1.80 | 0.88 | 1.00 | 0.96 | 0.78 | NA | NA | 1.35 | NA | 0.58 | 1.06 | NA | + | 8 | 2718 | 1.85 | 0.05 |
| 5 | 1.82 | 0.88 | NA | 1.07 | 0.78 | NA | NA | 1.35 | NA | 0.58 | 1.07 | 0.91 | + | 8 | 2718 | 1.97 | 0.05 |
| 6 | 1.83 | 0.87 | NA | 0.95 | 0.78 | NA | NA | 1.43 | 0.96 | 0.57 | 1.07 | NA | + | 8 | 2718 | 1.98 | 0.05 |
| 7 | 1.78 | 0.88 | 0.99 | 0.83 | 0.85 | 0.99 | 1.04 | 1.34 | NA | 0.58 | 1.06 | 0.90 | + | 11 | 2719 | 2.26 | 0.04 |
| 8 | 1.79 | 0.88 | 0.99 | 0.73 | 0.85 | 0.99 | 1.04 | 1.42 | 0.96 | 0.58 | 1.06 | NA | + | 11 | 2719 | 2.29 | 0.04 |
| 9 | 1.55 | 0.94 | 0.99 | 0.73 | 0.86 | 0.99 | 1.04 | 1.32 | NA | 0.67 | NA | NA | + | 9 | 2720 | 3.15 | 0.03 |
| 10 | 1.57 | 0.94 | NA | 0.96 | 0.78 | NA | NA | 1.33 | NA | 0.67 | NA | NA | + | 6 | 2720 | 3.16 | 0.03 |
| 11 | 1.77 | 0.88 | NA | 0.93 | NA | 0.96 | NA | 1.35 | NA | 0.59 | 1.06 | NA | + | 7 | 2720 | 3.25 | 0.02 |
| 12 | 1.78 | 0.88 | NA | NA | NA | NA | NA | 1.35 | NA | 0.58 | 1.06 | NA | + | 5 | 2720 | 3.30 | 0.02 |
| 13 | 1.79 | 0.88 | 0.99 | 0.92 | 0.81 | 0.98 | NA | 1.35 | NA | 0.58 | 1.06 | NA | + | 9 | 2720 | 3.32 | 0.02 |
| 14 | 1.81 | 0.88 | NA | 1.03 | 0.81 | 0.98 | NA | 1.35 | NA | 0.58 | 1.06 | 0.92 | + | 9 | 2720 | 3.47 | 0.02 |
| 15 | 1.82 | 0.88 | NA | 0.92 | 0.81 | 0.98 | NA | 1.43 | 0.96 | 0.58 | 1.07 | NA | + | 9 | 2720 | 3.48 | 0.02 |
| 16 | 1.81 | 0.88 | 1.00 | 1.07 | 0.78 | NA | NA | 1.35 | NA | 0.58 | 1.06 | 0.91 | + | 9 | 2720 | 3.82 | 0.02 |
| 17 | 1.82 | 0.87 | 1.00 | 0.95 | 0.78 | NA | NA | 1.44 | 0.95 | 0.57 | 1.07 | NA | + | 9 | 2720 | 3.83 | 0.02 |
| 18 | 1.83 | 0.88 | NA | 1.07 | 0.78 | NA | NA | 1.43 | 0.96 | 0.57 | 1.07 | 0.91 | + | 9 | 2720 | 3.95 | 0.02 |

Supplementary Table S3: Young birds show treatment specific associations between DTR and bird mortality (as indicated by the grey column).

| **Data Young** | **Temperature variables [°C]** | | | **Foraging treatment variables [Hard]** | | | | **Age related correction variables [Years]** | | | | | **Random term** | **Model Fit** | | | |
| --- | --- | --- | --- | --- | --- | --- | --- | --- | --- | --- | --- | --- | --- | --- | --- | --- | --- |
| **Model** | **DTR** | **MinT** | **DTR*MinT** | **Treat** | **Treat*DTR** | **Treat*MinT** | **Treat*DTR*MinT** | **AgeStart** | **AgeStart*Age** | **DTR*Age** | **MinT*Age** | **Treat*Age** | **Aviary** | **df** | **AICc** | **ΔAICc** | **weight** |
| 1 | 1.22 | 0.91 | 1.00 | 0.68 | 1.02 | 1.01 | 1.11 | 1.22 | NA | NA | NA | NA | + | 8 | 1191 | 0.00 | 0.08 |
| 2 | 1.20 | 0.91 | 1.00 | 0.67 | 1.02 | 1.01 | 1.11 | 0.74 | 1.97 | NA | NA | NA | + | 9 | 1192 | 0.65 | 0.06 |
| 3 | 1.20 | 0.86 | 1.00 | 0.68 | 1.01 | 1.01 | 1.11 | 1.22 | NA | NA | 1.08 | NA | + | 9 | 1192 | 0.80 | 0.06 |
| 4 | 1.21 | 0.91 | 1.00 | 1.25 | 1.05 | 1.01 | 1.12 | 1.22 | NA | NA | NA | 0.41 | + | 9 | 1192 | 1.06 | 0.05 |
| 5 | 1.18 | 0.86 | 1.01 | 0.68 | 1.01 | 1.01 | 1.11 | 0.69 | 2.12 | NA | 1.09 | NA | + | 10 | 1192 | 1.17 | 0.05 |
| 6 | 1.53 | 0.84 | 1.01 | 0.68 | 1.00 | 1.01 | 1.11 | 1.23 | NA | 0.70 | 1.13 | NA | + | 10 | 1192 | 1.22 | 0.05 |
| 7 | 1.35 | 0.91 | 1.00 | 0.67 | 1.02 | 1.01 | 1.11 | 1.23 | NA | 0.86 | NA | NA | + | 9 | 1193 | 1.62 | 0.04 |
| 8 | 1.20 | 0.91 | 1.00 | 1.27 | 1.04 | 1.01 | 1.12 | 0.73 | 2.01 | NA | NA | 0.40 | + | 10 | 1193 | 1.63 | 0.04 |
| 9 | 1.19 | 0.87 | 1.00 | 1.22 | 1.04 | 1.01 | 1.12 | 1.22 | NA | NA | 1.08 | 0.44 | + | 10 | 1193 | 1.98 | 0.03 |
| 10 | 1.18 | 0.86 | 1.00 | 1.24 | 1.03 | 1.02 | 1.12 | 0.69 | 2.16 | NA | 1.09 | 0.43 | + | 11 | 1193 | 2.29 | 0.03 |
| 11 | 1.54 | 0.84 | 1.01 | 1.23 | 1.01 | 1.02 | 1.12 | 1.23 | NA | 0.69 | 1.13 | 0.43 | + | 11 | 1193 | 2.35 | 0.03 |
| 12 | 1.41 | 0.85 | 1.01 | 0.68 | 1.00 | 1.02 | 1.11 | 0.82 | 1.71 | 0.78 | 1.13 | NA | + | 11 | 1194 | 2.55 | 0.02 |
| 13 | 1.23 | 0.91 | 1.00 | 0.67 | 1.01 | 1.01 | 1.11 | 0.75 | 1.91 | 0.97 | NA | NA | + | 10 | 1194 | 2.64 | 0.02 |
| 14 | 1.36 | 0.91 | 1.00 | 1.26 | 1.04 | 1.01 | 1.12 | 1.23 | NA | 0.85 | NA | 0.41 | + | 10 | 1194 | 2.64 | 0.02 |
| 15 | 1.24 | 0.91 | NA | NA | NA | NA | NA | 1.25 | NA | NA | NA | NA | + | 3 | 1194 | 2.89 | 0.02 |
| 16 | 1.22 | 0.86 | NA | NA | NA | NA | NA | 1.25 | NA | NA | 1.09 | NA | + | 4 | 1194 | 3.42 | 0.02 |
| 17 | 1.22 | 0.91 | NA | NA | NA | NA | NA | 0.76 | 1.98 | NA | NA | NA | + | 4 | 1194 | 3.50 | 0.01 |
| 18 | 1.23 | 0.91 | 1.00 | 1.27 | 1.04 | 1.01 | 1.12 | 0.75 | 1.93 | 0.96 | NA | 0.40 | + | 11 | 1195 | 3.61 | 0.01 |
| 19 | 1.41 | 0.85 | 1.01 | 1.25 | 1.01 | 1.02 | 1.12 | 0.81 | 1.74 | 0.77 | 1.12 | 0.42 | + | 12 | 1195 | 3.65 | 0.01 |
| 20 | 1.20 | 0.85 | NA | NA | NA | NA | NA | 0.70 | 2.16 | NA | 1.10 | NA | + | 5 | 1195 | 3.68 | 0.01 |
| 21 | 1.55 | 0.84 | NA | NA | NA | NA | NA | 1.27 | NA | 0.70 | 1.14 | NA | + | 5 | 1195 | 3.88 | 0.01 |

Supplementary Table S4: Old birds do not show treatment specific associations between DTR and bird mortality (as indicated by the grey column Treat*DTR*MinT). DTR overall has a positive effect on survival (grey column DTR).

| **Data Old** | **Temperature variables [°C]** | | | **Foraging treatment variables [Hard]** | | | | **Age related correction variables [Years]** | | | | | **Random term** | **Model Fit** | | | |
| --- | --- | --- | --- | --- | --- | --- | --- | --- | --- | --- | --- | --- | --- | --- | --- | --- | --- |
| **Model** | **DTR** | **MinT** | **DTR*MinT** | **Treat** | **Treat*DTR** | **Treat*MinT** | **Treat*DTR*MinT** | **AgeStart** | **AgeStart*Age** | **DTR*Age** | **MinT*Age** | **Treat*Age** | **Aviary** | **df** | **AICc** | **ΔAICc** | **weight** |
| 1 | 0.74 | 1.38 | NA | 0.82 | NA | 0.92 | NA | 1.40 | NA | NA | 0.82 | NA | + | 6 | 1534 | 0.00 | 0.07 |
| 2 | 0.73 | 1.35 | NA | 0.82 | NA | 0.92 | NA | 3.73 | 0.57 | NA | 0.83 | NA | + | 7 | 1534 | 0.37 | 0.06 |
| 3 | 0.74 | 1.36 | NA | 0.34 | NA | 0.93 | NA | 1.40 | NA | NA | 0.83 | 1.68 | + | 7 | 1535 | 1.51 | 0.03 |
| 4 | 0.74 | 1.38 | NA | 0.80 | 0.91 | 0.93 | NA | 1.40 | NA | NA | 0.82 | NA | + | 7 | 1535 | 1.67 | 0.03 |
| 5 | 0.74 | 1.39 | 0.99 | 0.82 | NA | 0.92 | NA | 1.40 | NA | NA | 0.82 | NA | + | 7 | 1535 | 1.74 | 0.03 |
| 6 | 0.55 | 1.45 | NA | 0.82 | NA | 0.92 | NA | 1.41 | NA | 1.19 | 0.80 | NA | + | 7 | 1536 | 1.84 | 0.03 |
| 7 | 0.73 | 1.34 | NA | 0.36 | NA | 0.93 | NA | 3.68 | 0.57 | NA | 0.84 | 1.64 | + | 8 | 1536 | 1.94 | 0.03 |
| 8 | 0.73 | 1.35 | NA | 0.79 | 0.91 | 0.93 | NA | 3.73 | 0.57 | NA | 0.83 | NA | + | 8 | 1536 | 2.03 | 0.03 |
| 9 | 0.73 | 1.37 | 0.99 | 0.81 | NA | 0.92 | NA | 3.79 | 0.56 | NA | 0.83 | NA | + | 8 | 1536 | 2.04 | 0.03 |
| 10 | 0.48 | 1.46 | NA | 0.82 | NA | 0.92 | NA | 4.00 | 0.55 | 1.28 | 0.80 | NA | + | 8 | 1536 | 2.06 | 0.03 |
| 11 | 0.74 | 1.37 | NA | 0.80 | 0.76 | NA | NA | 1.39 | NA | NA | 0.83 | NA | + | 6 | 1537 | 2.89 | 0.02 |
| 12 | 0.74 | 1.36 | NA | 0.32 | 0.91 | 0.94 | NA | 1.40 | NA | NA | 0.83 | 1.73 | + | 8 | 1537 | 3.13 | 0.02 |
| 13 | 0.73 | 1.34 | NA | 0.80 | 0.76 | NA | NA | 3.72 | 0.57 | NA | 0.84 | NA | + | 7 | 1537 | 3.23 | 0.01 |
| 14 | 0.74 | 1.38 | 0.99 | 0.34 | NA | 0.92 | NA | 1.40 | NA | NA | 0.82 | 1.67 | + | 8 | 1537 | 3.26 | 0.01 |
| 15 | 0.55 | 1.44 | NA | 0.34 | NA | 0.93 | NA | 1.41 | NA | 1.20 | 0.80 | 1.70 | + | 8 | 1537 | 3.34 | 0.01 |
| 16 | 0.74 | 1.39 | 0.99 | 0.79 | 0.92 | 0.93 | NA | 1.40 | NA | NA | 0.82 | NA | + | 8 | 1537 | 3.44 | 0.01 |
| 17 | 0.55 | 1.45 | NA | 0.80 | 0.91 | 0.93 | NA | 1.41 | NA | 1.20 | 0.80 | NA | + | 8 | 1537 | 3.50 | 0.01 |
| 18 | 0.73 | 1.34 | NA | 0.33 | 0.91 | 0.94 | NA | 3.68 | 0.57 | NA | 0.84 | 1.68 | + | 9 | 1537 | 3.56 | 0.01 |
| 19 | 0.56 | 1.46 | 0.99 | 0.82 | NA | 0.92 | NA | 1.41 | NA | 1.18 | 0.79 | NA | + | 8 | 1537 | 3.60 | 0.01 |
| 20 | 0.74 | 1.36 | NA | 0.20 | 0.77 | NA | NA | 1.39 | NA | NA | 0.83 | 2.27 | + | 7 | 1537 | 3.61 | 0.01 |
| 21 | 0.48 | 1.45 | NA | 0.35 | NA | 0.93 | NA | 3.96 | 0.55 | 1.29 | 0.80 | 1.65 | + | 9 | 1537 | 3.62 | 0.01 |
| 22 | 0.73 | 1.35 | 0.99 | 0.36 | NA | 0.92 | NA | 3.75 | 0.57 | NA | 0.83 | 1.63 | + | 9 | 1537 | 3.63 | 0.01 |
| 23 | 0.72 | NA | NA | 0.80 | 0.76 | NA | NA | 5.33 | 0.46 | NA | NA | NA | + | 5 | 1537 | 3.68 | 0.01 |
| 24 | 0.48 | 1.46 | NA | 0.79 | 0.91 | 0.93 | NA | 4.01 | 0.55 | 1.29 | 0.80 | NA | + | 9 | 1537 | 3.71 | 0.01 |
| 25 | 0.73 | 1.36 | 0.99 | 0.79 | 0.92 | 0.93 | NA | 3.80 | 0.56 | NA | 0.83 | NA | + | 9 | 1537 | 3.73 | 0.01 |
| 26 | 0.49 | 1.47 | 0.99 | 0.81 | NA | 0.92 | NA | 4.06 | 0.54 | 1.27 | 0.79 | NA | + | 9 | 1537 | 3.76 | 0.01 |
| 27 | 1.41 | NA | NA | 0.80 | 0.76 | NA | NA | 4.08 | 0.53 | 0.66 | NA | NA | + | 6 | 1537 | 3.80 | 0.01 |
| 28 | 0.75 | 0.98 | NA | 0.82 | NA | 0.92 | NA | 5.28 | 0.47 | NA | NA | NA | + | 6 | 1538 | 3.95 | 0.01 |

**Supplementary material 3: Model support for the absence of sex specific effects.**

Supplementary Table S5: Weak support for sex-specific associations between DTR and bird mortality (as indicated by the grey column) in the easy foraging environment.

| **Easy treatment** | **Temperature variables [°C]** | | | **Sex specific variables [Male]** | | | **Age related correction variables [Years]** | | | | **Random term** | **Model Fit** | | | |
| --- | --- | --- | --- | --- | --- | --- | --- | --- | --- | --- | --- | --- | --- | --- | --- |
| **Model** | **DTR** | **MinT** | **DTR*MinT** | **Sex** | **DTR*Sex** | **Sex*Age** | **AgeStart** | **AgeStart*Age** | **DTR*Age** | **MinT*Age** | **Aviary** | **df** | **AICc** | **ΔAICc** | **weight** |
| 1 | 1.36 | 0.89 | 0.95 | NA | NA | NA | 1.25 | NA | 0.74 | 1.06 | + | 10 | 1269 | 0.00 | 0.06 |
| 2 | 1.22 | 0.95 | 0.95 | NA | NA | NA | 1.24 | NA | 0.83 | NA | + | 9 | 1269 | 0.03 | 0.06 |
| 3 | 0.98 | 0.95 | 0.95 | 1.63 | NA | 0.59 | 1.22 | NA | NA | NA | + | 10 | 1269 | 0.42 | 0.05 |
| 4 | 0.98 | 0.95 | 0.95 | NA | NA | NA | 1.22 | NA | NA | NA | + | 8 | 1270 | 0.65 | 0.05 |
| 5 | 1.22 | 0.95 | 0.95 | 1.63 | NA | 0.59 | 1.23 | NA | 0.83 | NA | + | 11 | 1270 | 0.68 | 0.05 |
| 6 | 1.36 | 0.89 | 0.95 | 1.63 | NA | 0.59 | 1.25 | NA | 0.75 | 1.06 | + | 12 | 1270 | 0.88 | 0.04 |
| 7 | 0.98 | 0.95 | 0.95 | 0.78 | NA | NA | 1.22 | NA | NA | NA | + | 9 | 1270 | 1.31 | 0.03 |
| 8 | 1.40 | 0.88 | 0.95 | NA | NA | NA | 1.69 | 0.81 | 0.73 | 1.07 | + | 11 | 1270 | 1.35 | 0.03 |
| 9 | 1.22 | 0.95 | 0.95 | 0.79 | NA | NA | 1.24 | NA | 0.83 | NA | + | 10 | 1270 | 1.52 | 0.03 |
| 10 | 1.24 | 0.95 | 0.95 | NA | NA | NA | 1.56 | 0.85 | 0.83 | NA | + | 10 | 1271 | 1.62 | 0.03 |
| 11 | 0.98 | 0.95 | 0.95 | 1.70 | 1.11 | 0.58 | 1.21 | NA | NA | NA | + | 11 | 1271 | 1.67 | 0.03 |
| 12 | 0.97 | 0.91 | 0.95 | 1.63 | NA | 0.59 | 1.22 | NA | NA | 1.03 | + | 11 | 1271 | 1.79 | 0.03 |
| 13 | 1.20 | 0.95 | 0.95 | 1.67 | 1.10 | 0.59 | 1.23 | NA | 0.84 | NA | + | 12 | 1271 | 1.90 | 0.02 |
| 14 | 1.35 | 0.89 | 0.95 | 1.67 | 1.10 | 0.59 | 1.25 | NA | 0.75 | 1.06 | + | 13 | 1271 | 2.03 | 0.02 |
| 15 | 0.97 | 0.91 | 0.95 | NA | NA | NA | 1.23 | NA | NA | 1.03 | + | 9 | 1271 | 2.08 | 0.02 |
| 16 | 0.98 | 0.95 | 0.95 | 1.63 | NA | 0.59 | 1.49 | 0.87 | NA | NA | + | 11 | 1271 | 2.12 | 0.02 |
| 17 | 1.36 | 0.89 | 0.95 | 0.78 | NA | NA | 1.25 | NA | 0.74 | 1.06 | + | 11 | 1271 | 2.20 | 0.02 |
| 18 | 1.40 | 0.88 | 0.95 | 1.63 | NA | 0.59 | 1.70 | 0.80 | 0.73 | 1.07 | + | 13 | 1271 | 2.24 | 0.02 |
| 19 | 1.24 | 0.95 | 0.95 | 1.63 | NA | 0.59 | 1.57 | 0.85 | 0.83 | NA | + | 12 | 1271 | 2.26 | 0.02 |
| 20 | 0.98 | 0.95 | 0.95 | NA | NA | NA | 1.47 | 0.88 | NA | NA | + | 9 | 1271 | 2.39 | 0.02 |
| 21 | 1.22 | 0.95 | 0.95 | 0.81 | 1.10 | NA | 1.24 | NA | 0.83 | NA | + | 11 | 1272 | 2.67 | 0.02 |
| 22 | 0.98 | 0.95 | 0.95 | 0.80 | 1.10 | NA | 1.22 | NA | NA | NA | + | 10 | 1272 | 2.74 | 0.02 |
| 23 | 0.97 | 0.91 | 0.95 | 0.78 | NA | NA | 1.23 | NA | NA | 1.03 | + | 10 | 1272 | 2.75 | 0.02 |
| 24 | 1.37 | 0.89 | 0.95 | 0.81 | 1.10 | NA | 1.26 | NA | 0.74 | 1.06 | + | 12 | 1272 | 2.78 | 0.02 |
| 25 | 0.97 | 0.91 | 0.95 | 1.71 | 1.11 | 0.58 | 1.22 | NA | NA | 1.03 | + | 12 | 1272 | 2.97 | 0.01 |
| 26 | 0.98 | 0.95 | 0.95 | 0.78 | NA | NA | 1.50 | 0.87 | NA | NA | + | 10 | 1272 | 3.01 | 0.01 |
| 27 | 1.24 | 0.95 | 0.95 | 0.78 | NA | NA | 1.57 | 0.85 | 0.83 | NA | + | 11 | 1272 | 3.14 | 0.01 |
| 28 | 0.98 | 0.95 | 0.95 | 1.70 | 1.11 | 0.58 | 1.50 | 0.86 | NA | NA | + | 12 | 1272 | 3.35 | 0.01 |
| 29 | 1.39 | 0.88 | 0.95 | 1.67 | 1.10 | 0.59 | 1.71 | 0.80 | 0.74 | 1.07 | + | 14 | 1272 | 3.40 | 0.01 |
| 30 | 0.97 | 0.91 | 0.95 | 1.63 | NA | 0.59 | 1.54 | 0.85 | NA | 1.04 | + | 12 | 1272 | 3.42 | 0.01 |
| 31 | 1.22 | 0.95 | 0.95 | 1.67 | 1.10 | 0.59 | 1.57 | 0.84 | 0.83 | NA | + | 13 | 1272 | 3.51 | 0.01 |
| 32 | 1.41 | 0.88 | 0.95 | 0.78 | NA | NA | 1.70 | 0.81 | 0.73 | 1.07 | + | 12 | 1273 | 3.59 | 0.01 |
| 33 | NA | 0.95 | NA | 1.61 | NA | 0.59 | 1.21 | NA | NA | NA | + | 8 | 1273 | 3.71 | 0.01 |
| 34 | 0.97 | 0.91 | 0.95 | NA | NA | NA | 1.52 | 0.86 | NA | 1.04 | + | 10 | 1273 | 3.75 | 0.01 |

Supplementary Table S6: No sex-specific effects in the association between DTR and bird mortality (as indicated by the grey column) in the hard foraging environment.

| **Hard treatment** | **Temperature variables [°C]** | | | **Development** | **Sex specific variables [Male]** | | | **Age related correction variables [Years]** | | | | **Random term** | **Model Fit** | | | |
| --- | --- | --- | --- | --- | --- | --- | --- | --- | --- | --- | --- | --- | --- | --- | --- | --- |
| **Model** | **DTR** | **MinT** | **DTR*MinT** | **Brood size** | **Sex** | **DTR*Sex** | **Sex*Age** | **AgeStart** | **AgeStart*Age** | **DTR*Age** | **MinT*Age** | **Aviary** | **df** | **AICc** | **ΔAICc** | **weight** |
| 1 | 2.04 | 0.88 | 1.03 | 1.15 | NA | NA | NA | 1.34 | NA | 0.50 | 1.05 | + | 11 | 1247 | 0.00 | 0.50 |
| 2 | 1.99 | 0.88 | 1.03 | 1.15 | NA | NA | NA | 1.16 | 1.12 | 0.50 | 1.05 | + | 12 | 1249 | 1.70 | 0.21 |
| 3 | 1.82 | 0.93 | 1.04 | 1.16 | NA | NA | NA | 1.30 | NA | 0.56 | NA | + | 10 | 1252 | 5.31 | 0.04 |

**Supplementary material 4: Age specific DTR effects**

Supplementary Figure S3: Associations between DTR and hazard rate change with age for the easy foraging environment (top graphs in white) and semi-natural foraging environment (lower graphs in grey). From left to right: fitted lines for the ages of 0.36, 1.65, 4.00 and 5.07 years (at which 90%, 70%, 30% and 10% of the population was alive respectively). Lines within each graph show days with different minimum temperatures, indicated by numbers next to each line. Grey horizontal lines are reference lines at hazard rates of 0.1, 1 and 10. Graphs can be interpreted as follows: (i) In the easy foraging environment, young birds suffer high mortality when cold days get warmer (large DTR). In contrast, old birds remain unaffected by DTR on cold days, but do best when warm days get warmer. (ii) In the semi-natural foraging environment, high mortality occurs for young birds when warm days get warm (large DTR), but for old birds days that do not get warm are most lethal (small DTR). This suggests that DTR affects mortality via different ways in young versus old birds. For example, old birds in the semi-natural foraging environment, the DTR effect is likely due to cold exposure.

Hazard rate

Easy environment

Semi-natural environment

Diurnal temperature range [°C]

.

.

.

.

.

.

.

.

Age=0.36 years (90% alive)

Age=1.65 years (70% alive)

Age=4 years (30% alive)

Age=5.06 years (10% alive)

**Supplementary material 5: Isolines representation of association between DTR and mortality**

Supplementary
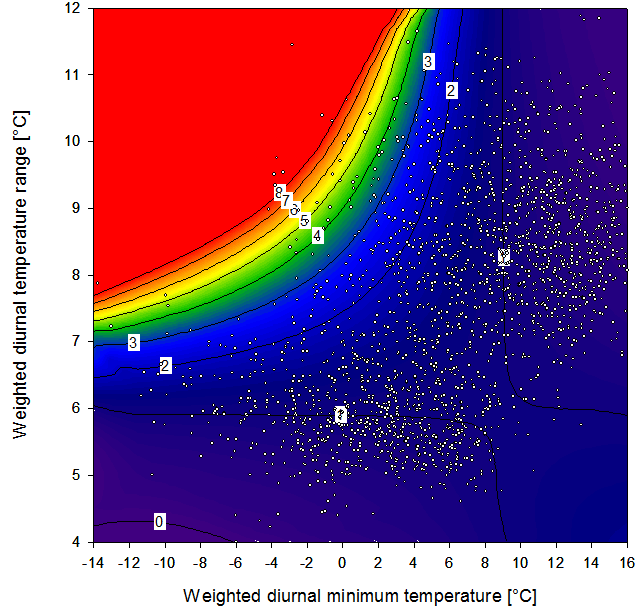
 Figure S4: Effects of natural variation in minimum temperature and diurnal temperature range on the hazard rate of zebra finches in easy foraging treatment (A) and semi natural environment (B). Isolines connect data with the same relative hazard rate and are the result of the model (Table 1, calculated for the age of 0.36 years as in fig. 4) which is based on the daily observation of survival of 229 (A) and 246 (B) individuals from December 9^th^ 2007 till January 1^st^ 2013. Grey dots represent weighted weather data.

A

B


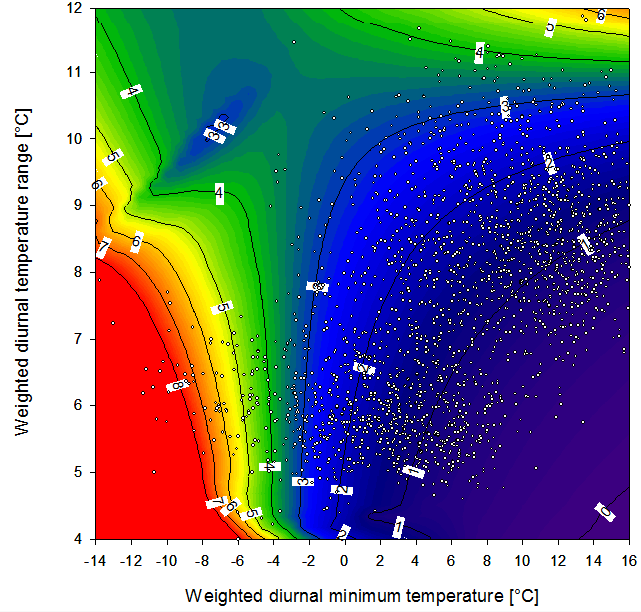

Supplement: Supplementary Information [file srep16600-s1.doc]
